# Allelopathic Effects of Water Hyacinth [*Eichhornia crassipes*]

**DOI:** 10.1371/journal.pone.0013200

**Published:** 2010-10-08

**Authors:** Sanaa M. M. Shanab, Emad A. Shalaby, David A. Lightfoot, Hany A. El-Shemy

**Affiliations:** 1 Botany Department, Faculty of Science, Cairo University, Giza, Egypt; 2 Biochemistry Department, Faculty of Agriculture, Cairo University, Giza, Egypt; 3 Genomics Core-Facility, Southern Illinois University at Carbondale, Carbondale, Illinois, United States of America; University of Hyderabad, India

## Abstract

*Eichhornia crassipes* (Mart) Solms is an invasive weed known to out-compete native plants and negatively affect microbes including phytoplankton. The spread and population density of *E. crassipes* will be favored by global warming. The aim here was to identify compounds that underlie the effects on microbes. The entire plant of *E. crassipes* was collected from El Zomor canal, River Nile (Egypt), washed clean, then air dried. Plant tissue was extracted three times with methanol and fractionated by thin layer chromatography (TLC). The crude methanolic extract and five fractions from TLC (A–E) were tested for antimicrobial (bacteria and fungal) and anti-algal activities (green microalgae and cyanobacteria) using paper disc diffusion bioassay. The crude extract as well as all five TLC fractions exhibited antibacterial activities against both the Gram positive bacteria; *Bacillus subtilis and Streptococcus faecalis*; and the Gram negative bacteria; *Escherichia coli and Staphylococcus aureus*. Growth of *Aspergillus flavus* and *Aspergillus niger* were not inhibited by either *E. crassipes* crude extract nor its five fractions. In contrast, *Candida albicans* (yeast) was inhibited by all. Some antialgal activity of the crude extract and its fractions was manifest against the green microalgae; *Chlorella vulgaris* and *Dictyochloropsis splendida* as well as the cyanobacteria; *Spirulina platensis* and *Nostoc piscinale*. High antialgal activity was only recorded against *Chlorella vulgaris*. Identifications of the active antimicrobial and antialgal compounds of the crude extract as well as the five TLC fractions were carried out using gas chromatography combined with mass spectroscopy. The analyses showed the presence of an alkaloid (fraction A) and four phthalate derivatives (Fractions B–E) that exhibited the antimicrobial and antialgal activities.

## Introduction

Water hyacinth, *Eichhornia crassipes* (Mart) Solms, originated in the state of Amazon, Brazil, spread to other regions of South America, and was carried by humans throughout the tropics and subtropics. It is now widespread and recognized as one of the top 10 weeds in the world. Water hyacinth has invaded Africa, Asia, North America and will occur in at least 62 countries by 2010. It causes extremely serious ecological, economical and social problems in regions between 40 degrees north and 45 degree south [1].

*E. crassipes* forms dense monocultures that can threaten local native species diversity and change the physical and chemical aquatic environment, thus altering ecosystem structure and function by disrupting food chains and nutrient cycling. The large, dense monoculture formed by this species covers lakes and rivers, blocking waterways and interfering with the water transport of agriculture products, tourism activities, water power and irrigation of agricultural fields. Dense mats of water hyacinth can lower dissolved oxygen levels in water bodies leading to reduction of aquatic fish production. Water hyacinth is very efficient in taking up Calcium, Magnesium, Sulfur, Ferric, Manganese, Aluminum, Boron, Cupper, Molybdenum, Zinc, Nitrogen, Phosphorus and potassium favoring its growth over other aquatic species [2].

When this macrophyte (water hyacinth) dies, sinks and decomposes, the water becomes more eutrophic due to the large release of nutrients [1]. Water quality deteriorated, clean drinking water can be threatened and human health impacted. Aggressive growth of *E. crassipes* was correlated with increased temperature, high solar radiation and sunshine duration which may result in an intensive plant growth during summer that may be increased by global warming. High biomass production of water hyacinth corresponded with large amounts of phenolic allelochemicals in the water, which may also help in the process of invasion. High air temperatures in summer (>35°C) caused an increase in the rate of evapo-transpiration leading to decrease in water level and consequently a possible increase in allelochemical concentration in the aquatic habitats [2].

Among the consequent serious problems of invasion by water hyacinth is the vast range and rapid spread of the aquatic weeds in the Egyptian water bodies, particularly in the network of irrigation and drainage canals in the Nile Delta region [3]. It produces serious problems due to increased water loss and evaporation, retardation of water flow, interference with navigation, health hazards and alteration in the physicochemical characteristics of both water and hydrosoil [4]. The major effort in controlling *E. crassipes* in Egypt is focused on mechanical removal, but infestations soon returned [5]. The removal and accumulation of the dead plant materials that contain allelochemicals on canal banks and the dissolving of these allelochemicals in irrigation waters led to inhibition of seed germination as well as growth of crops and economic plants. Macrophytes [hydrophytic plants and pteridophytes] and algae were known to have antagonistic relationships in both natural and experimental aquatic ecosystems [6]. It was reported that, macrophytes both release and accumulate bioactive allelochemical secondary metabolites in quantities sufficient to inhibit algal growth [7]–[16]. In return, algae exert an inhibitory effect on macrophytes not only by competing with available nutrients supply, but also by secreting substances with herbicidal activity [17], [18].

Complex interactive effects were reported between the invasive plants growth (*E. crassipes*), the occurrence of allelochemicals (phenolic compounds) and biotic and abiotic environmental factors [[19] and [20]]. Chromatographic and spectroscopic analyses showed that macrophytes can release fatty acids, steroids, polyphenols and tannins [11]–[13], [21]–[22], [23]. Many studies were carried out on water hyacinth concerning either its growth inhibition by the coexisting microalgae in the same ecosystem [17], [18] or its antialgal activity manifested on many algal species belonging to different algal divisions [20], [24], [25], [26], . However, little was known by 2010 about the the active compounds that responsible for these effects. Only Jin et al. and Awad isolated allelochemicals from crude *E. crassipes* extracts by fractionation which exerted allelopathic effect on the green algae tested and inhibited the germination of seeds and growth of seedlings of some crop plants [4], [27]. The allelochemicals identified by Awad [4] were found to be chloro- and nitro-phenols.

This work aimed to study the influence of methanolic extracts and their fractions from *E. crassipes* on microorganisms [pathogenic bacteria and fungi] as well as on the photoautotrophic phytoplanktons inhabiting the same aquatic ecosystyems (microalgae and cyanobacteria) using the paper disc diffusion bioassay.

## Materials and Methods

### Chemicals and reagents

Pure hexane, chloroform, ethanol, ether, acetone, methanol and acetic acid were purchased from E. Merck Co. (Darmstadt, Germany), and distilled before use. Tetracycline [antibacterial agent] and amphotericin B (antifungal agent) were obtained from Sigma Chemical Co. (St. Louis, MO, USA).

### Collection and extraction of water hyacinth

*E. crassipes* (Mart) Solms. was collected from the River Nile at the El-Zomor canal [Giza], Egypt, cleaned from any debris, washed several times with tap, distilled and then sterilized water, air dried (or lyophilized), ground in methanol and then stored at −20°C until use. A known weight (500g) of the macrophyte was extracted three times (with 24 hrs intervals) with methanol at room temperature and in the dark. The solvent of the combined extracts were evaporated, and solids concentrated, using rotary evaporator at 40°C according to Rossenthaler [29].

### GC/MS Analysis of crude extract

The crude methanolic extract of *Eichhornia crassipes* was analyzed by GC-MS. GC-MS analysis was performed on a Thermoquest- Finnigan Trace GC-MS equipped with a DB-5 (5% [w/v] phenyl) methylpolysiloxane column (60 m\0.25 mm i.d., film thickness 0.25 µm). The injection temperature was 220°C and the oven temperature was raised from 40°C [3 min hold] to 250°C at a rate of 5°C/min, then held at 250°C for 2 min; transfer line temperature was 250°C. One microliter of sample was injected and helium was used as the carrier gas at a flow rate of 1.0 ml/min. The mass spectrometer was scanned over the 40 to 500 m/z range with an ionizing voltage of 70 eV and identification was based on standard mass library of the National Institute of Standards and Technology (NIST Version 2.0) to detect the possible extract components.

### Fractionation of the crude extract

**Figure 1:**
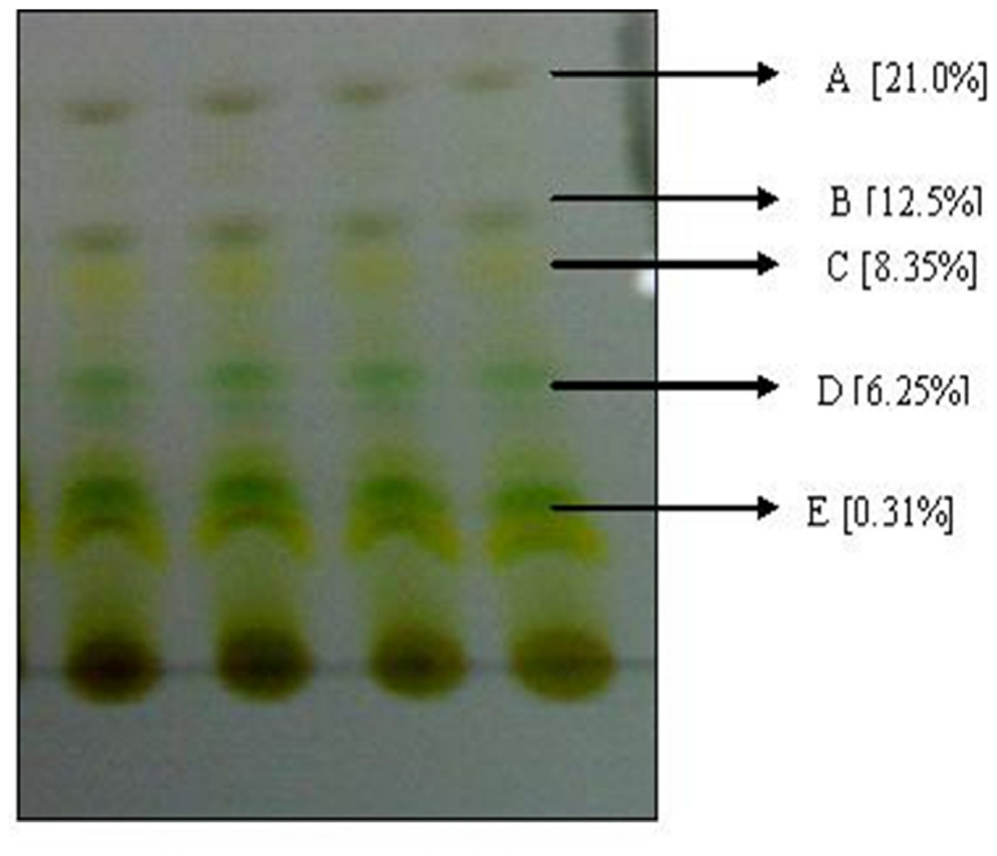
Fractionation of crude methanolic extract of *Eichhornia crassipes* using silica gel TLC. Using hexane/ethyl acetate (8.5∶1.5, v/v) as mobile phase.

Using pre-coated TLC F_254_ plates, the crude extract was fractionated using different combinations of hexane/ethyl acetate solvents (9∶1, 8.5∶1.5, 8∶2, 7∶3 and 5∶5) as the mobile phase. The separated fractions (A–E) by the mixture hexane/ethyl acetate (8.5∶1.5, v/v) which seemed the best were scratched and eluted with the same mobile phase, filtered, evaporated, weighed and then used in bioassays (Fig 1).

The most potent fractions were chosen for further identification using the chromatographic and spectroscopic methods as follows.

### a-Mass spectroscopic (MS) Analysis of potent fractions

The potent fractions of *E. crassipes* were analyzed by Mass spectrum (MS). The mass spectrometer was scanned over the 40 to 500 m/z range with an ionizing voltage of 70 eV and identification was based on standard mass library that National Institute of Standards and Technology (NIST Version 2.0) to detect the possible fraction structure.

### b-Fourier transformed infra red (FTIR) spectra

A Perkin Elmer (Waltham, Massachusetts, USA) was used to obtain Fourier transformed infrared (FTIR) spectra (System 2000).

### c-Proton magnetic resonance Spectra (^1^H NMR)

The identification of compounds was confirmed by carrying out ^1^H-NMR analysis using NMR Joel GIM, EX 270 (400 Hz).

### Screening for antimicrobial activity

The crude methanolic extract as well as the TLC separated fractions were tested for antimicrobial activity using four bacterial species (Gram positive bacteria: *Bacillus subtilis* and *Streptococcus faecalis*; Gram negative bacteria: *Escherichia coli* and *Staphylococcus aureus*), two fungal species (*A. flavus* and *A. niger*) and one yeast (*C. albicans*). Strains were obtained from Microbiology Department, Microanalytical Center, Faculty of Science, Cairo University and they will be available on request. Antimicrobial activity was screened by using the paper disc diffusion bioassay, and the diameter of inhibition zones were compared with those obtained by the standard antibacterial agent; tetracycline and amphotericin (antifungal agent B).

Sterilized filter paper discs (6 mm) saturated with solutions of either tetracycline, crude extract or fraction(s) thereof at 20 to 250 µg/ml were placed on the surface of Petri dishes (14 cm) containing solid bacterial medium [nutrient agar broth] Fungal Doxs medium seeded with cell or spore suspensions of the fungal species and standard antifungal amphotericin B were also performed. The inoculated plates were incubated in the favorable conditions for bacterial (35–37°C for 24–48 hrs) and fungal growth (25–27°C for 3–7 days). The diameter of the clear inhibition zones surrounding the paper disc saturated with the crude methanolic extract or hexane/ethyl acetate fractions were taken as a measure of the inhibitory power of the sample against the particular test organisms [30]–[32]. Standard antibacterial and antifungal agents were used as positive controls (tetracycline and amphotericin B) respectively. All experiments were carried out in triplicates.

### Screening for antialgal activities

The crude methanolic extract and the TLC separated fractions were tested against two green microalgae (*Chlorella vulgaris* Beijer., *Dictyochloropsis splendida* Geitler.) and two cyanobacterial species (*Spirulina platensis* (Nordist.) Geitler. and *Nostoc piscinale* Kutz.) Both were isolated (from River Nile), identified [33]–[36] and cultured by the first author. They are available on request. The filter papers were loaded with 20–250 µg/ml fractions or crude extract. The inoculated plates were overlaid with the dried filter papers at their center and incubated at the optimal conditions for algal growth (20±1°C, light intensity of 30 µE/m^2^/s and photoperiod of 16/8 light, dark cycles for 10 days). The diameter of the clear inhibition zones surrounding the paper discs saturated with crude extract or fractions after 24 or 72 h were taken as a measure of the inhibitory power of the extracts against each of the tested algal species. Minimum inhibitory concentration (MIC) was determined by paper disk agar diffusion methods according to Chattopadhyay et al. [37]. The lowest concentration which did not show any visible growth was considered as the MIC.

For identification of the bioactive substances in extract and fractions, methylation of the crude extract by ethereal diazomethane (CH_2_N_2_) was performed before injection in gas chromatography/Mass spectroscopy. The fractions were identified by spectroscopic methods (Microanalytical Center, Cairo University).

## Results and Discussion

The results in Fig. 1 and Table 1 shows that both crude methanolic extract (K) and the five thin layer chromatography (TLC) fractions (A–E) exhibit antibacterial activities of different percentages (21.0, 12.5, 8.35, 6.25 and 0.31%) respectively. Crude extract and fractions showed moderate activities against the Gram positive and Gram negative bacteria species. The activity represents nearly 50% of that recorded by the standard antibacterial agent tetracycline. The antibacterial activity measured by the diameter of inhibition zone surrounding the paper discs saturated with the crude extract was shown to be similar, slightly decreased or increased by the different TLC separated fractions. This suggests that the crude extract contained different antibacterial substances with variable efficiencies and mode of actions which may act antagonistically leading to a decrease in the diameter of inhibition zone (as in case of fraction C (8.35%) with *E. coli*) or synergistically causing an increase in the diameter of inhibition zone as in case of fractions A (21.0%) and B (12.5%) with *S. faecalis*, compared with the crude extract (K). Alternately, the greater purity of fractions may show the opposite effects of higher concentrations of active compounds offset by fewer bioactive compounds present.

**Table 1:** Diameter of inhibition zones (mm) of crude methanolic extract and fractions of *Eichhornia crassipes* against tested microorganisms (bacteria after 24h and fungi after 72h).

Diameter of inhibition zone (mm)										
Microorganism	Gram reaction	Standard Ab		Fractions						
		Tetra	Amph	Crude K	A	B	C	D	E	LSD at 0.01
*Bacillus subtilis*	+	33±0.0	–	12±0.5	13±0.0	14±0.3	12±0.0	14±0.5	13±0.2	0.25
*Escherichia coli*	−	33±1.5	–	12±0.0	14±1.0	14±0.0	11±0.5	13±0.3	12±0.4	0.25
*Staphylococcus aureus*	−	32±0.6	–	12±0.2	15±0.2	13±0.0	12±0.6	15±0.0	15±1.2	0.25
*Streptococcus faecalis*	+	30±0.9	–	14±0.2	16±0.7	15±1.0	12±0.0	14±0.8	13±0.5	0.25
*Aspergillus flavus*	Fungus	–	16±0.8	0±0.0	0±0.0	0±0.0	0±0.0	0±0.0	0±0.0	0.09
*Aspergillus niger*	Fungus	–	15±0.0	0±0.0	0±0.0	0±0.0	0±0.0	0±0.0	0±0.0	0.09
*Candida albicans*	Yeast	–	18±0.0	13±0.5	12±1.1	15±0.4	14±0.0	11±0.3	12±0.0	0.25
LSD at 0.01		0.326	0.413	0.21	0.21	0.21	0.21	0.21	0.21	

Abbreviation: Ab, antibiotic; Tetra, Tetracycline (antibacterial agent); Amph, Amphotericin B (antifungal agent).

*–: not tested; Each value is presented as mean of triplicate treatments, LSD: Least different significantly at p≤0.01 according to Duncan's multiple range test. Data are expressed as mean ± S.D.

Antifungal activities of extracts [crude and fractions] were manifested only against *C. albicans* (yeast). Both *A. flavus* and *A. niger* were shown to be highly resistant to all extracts and gave no sign of growth inhibition (Table 1). Fractions B and C, exhibited higher activities against *Candida albicans* than those of fractions A (21.0%), D (6.25%) and E (0.31%).

From all the tested algal species (2 green microalgae and 2 cyanobacteria), water hyacinth crude extract and fractions exhibit potent antialgal activity against only the green microalga *Chlorella vulgaris* (Table 2). The growth of other algal species demonstrated no sign of inhibition caused by all extracts.

**Table 2:** Diameter of inhibition zones (mm) around paper disc loaded with crude methanolic extract and fractions of *Eichhornia crassipes* against tested microalgae after 72–168h.

Fraction	Inhibition zone diameter (mm)			
	*Dictyo.*	*Nos.*	*Chl.*	*Spir.*
Crude (K)	0±0.0	0±0.0	13±0.2	0±0.0
A	0±0.0	0±0.0	33±1.5	0±0.0
B	0±0.0	0±0.0	22±0.8	0±0.0
C	0±0.0	0±0.0	18±0.0	0±0.0
D	0±0.0	0±0.0	26±0.6	0±0.0
E	0±0.0	0±0.0	31±1.8	0±0.0
LSD at 0.01	-	-	0.26	-

Abbreviation: Dictyo.  =  *Dictyochloropsis splendid*; Nos.  =  *Nostoc piscinale*; Chl.  =  *Chlorella vulgaris*; Spir.  =  *Spirulina platensis*.

*Each value is presented as mean of triplicate treatments, LSD: Least different significantly at p≤0.01 according to Duncan's multiple range test. Data are expressed as mean ± S.D.

Data from Table 1 and 2 illustrates clearly that the crude methanolic extract (K) showed a moderate zone of inhibition (13 mm) which greatly enlarged by the five fractions (18–33 mm) indicating the increase in activity. Fractions A manifested the greatest antialgal activity *against Chlorella vulgaris* (33 mm) followed in descending order by fractions E, D, B and C.

The lowest antialgal activity was exhibited by the crude methanolic extract and the greatest activity of the five fractions indicated that different antialgal substances may be present in the crude extract. Compounds in the crude extract might act antagonistically leading to a marked decrease in activity (inhibition zone of 13 mm) whereas, the separated and highly purified fractions showed greater activity manifested by an enlargement of inhibition zone (18–33 mm). Fractionation of this crude extract and the separation of these antialgal compounds in the form of the five fractions may have alleviated the antagonism between compounds in crude extract or raised the purity of these active compounds leading to increase in diameter of inhibition zone (18–33 mm).

Chromatographic and spectroscopic analysis of the crude extract and fractions suggested that, 1, 2-Benzenedicarboxylic acid bis (2-ethylhexyl) ester was present in both crude extract and fraction C. This compound has potent antibacterial, antifungal as well as moderate antialgal activities and as recorded by many investigators from different sources [38]–[40]. El-Mehalawy et al. [40] showed that this compound was produced by certain bacteria (*Tsukamurella inchonensis*, *Corynebacterium nitrilophilus* and *Cellulosimicrbium cellulans*). The cpmpound inhibited fungal spore germination, cell membrane growth and production of total lipid and total protein among six human and plant pathogenic fungi (*A. flavus*, *A. terreus*, *A.* niger, *Fusarium oxysporium*, *Altermaria solani* and *Penicillium digitutum*).

**Figure 2:**
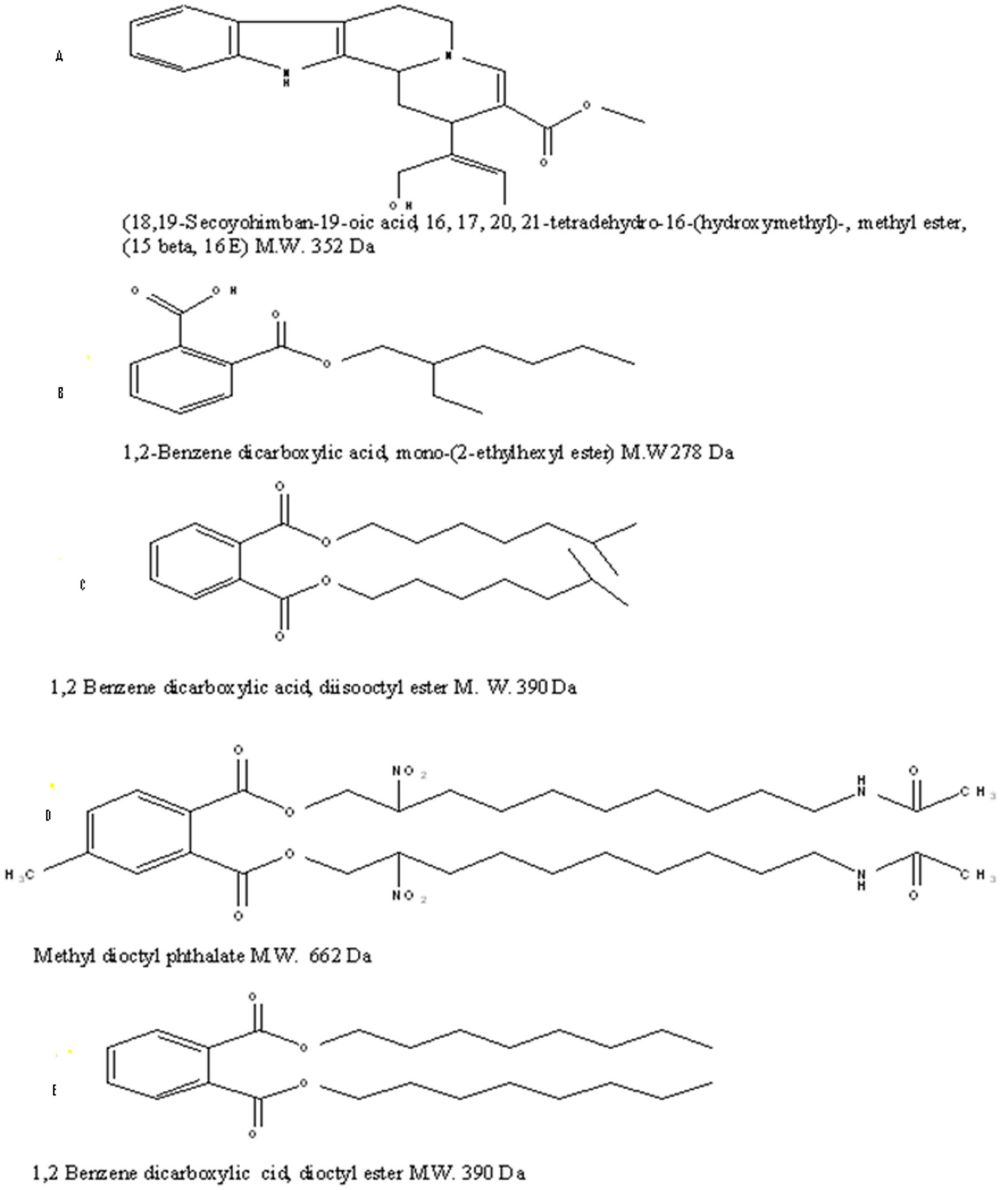
Predicted chemical structure of different active compounds separated from *Eichhornia crassipes*.

**Figure 3:**
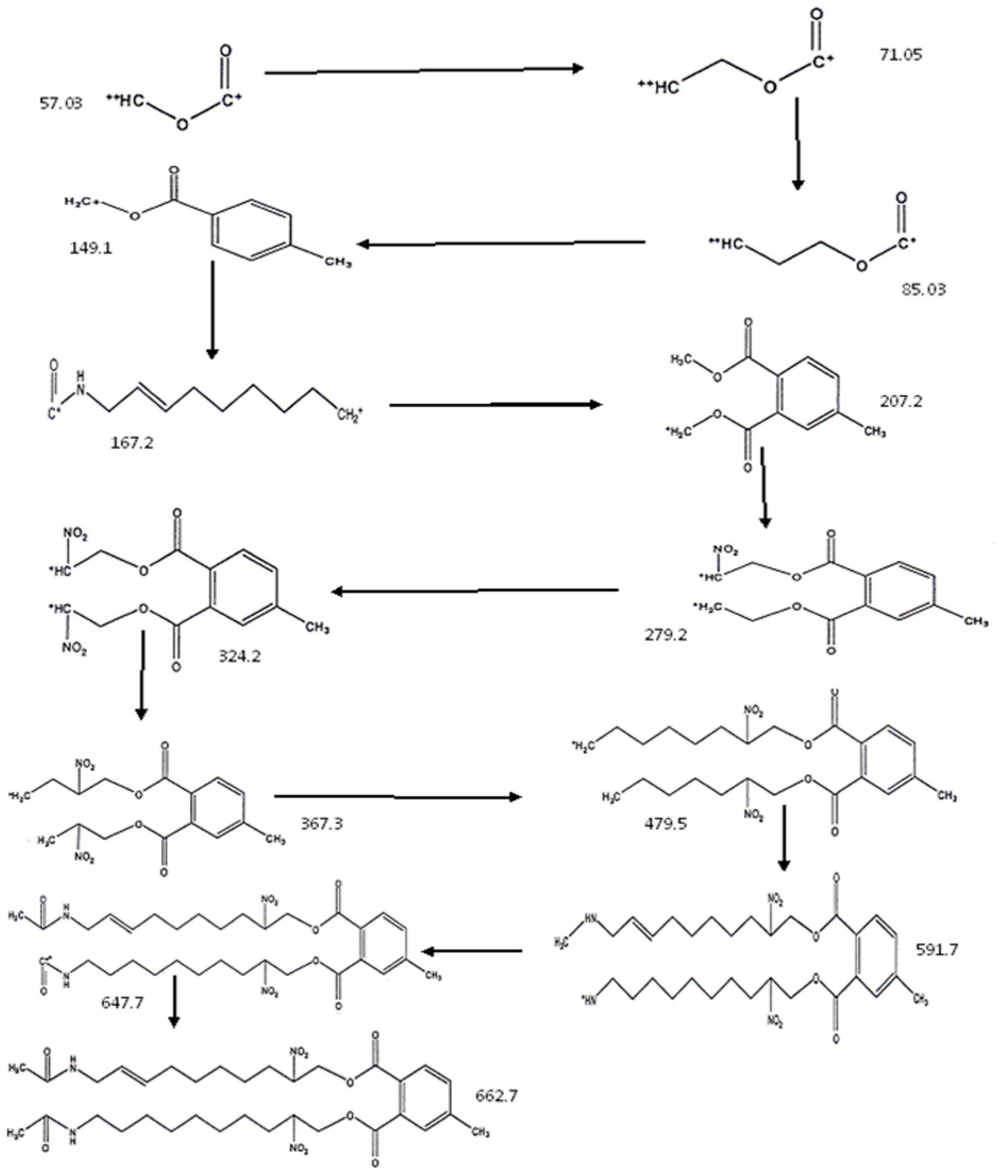
Fragmentation pattern of active ingredient (chemical structure and M. Wt) from *Eichhornia crassipes*.

Fractions B, D and E contained compounds that were identified by spectroscopic methods (Fig. 2 and Fig. 3) as phthalate derivatives (ethylhexyl, methyl-dioctyl and dioctyl phthalate with molecular weights 278, 662 and 390 respectively) which manifested moderate antimicrobial and antialgal activities (Table 1 and 2). These findings agreed with those reported for phthalate derivatives isolated from; seaweeds [41], [42]; marine sponges; and from bacteria [43]–[45]. Those phthalate derivatives exhibited moderate activities against different pathogenic bacteria, unicellular and filamentous fungi.

**Figure 4:**
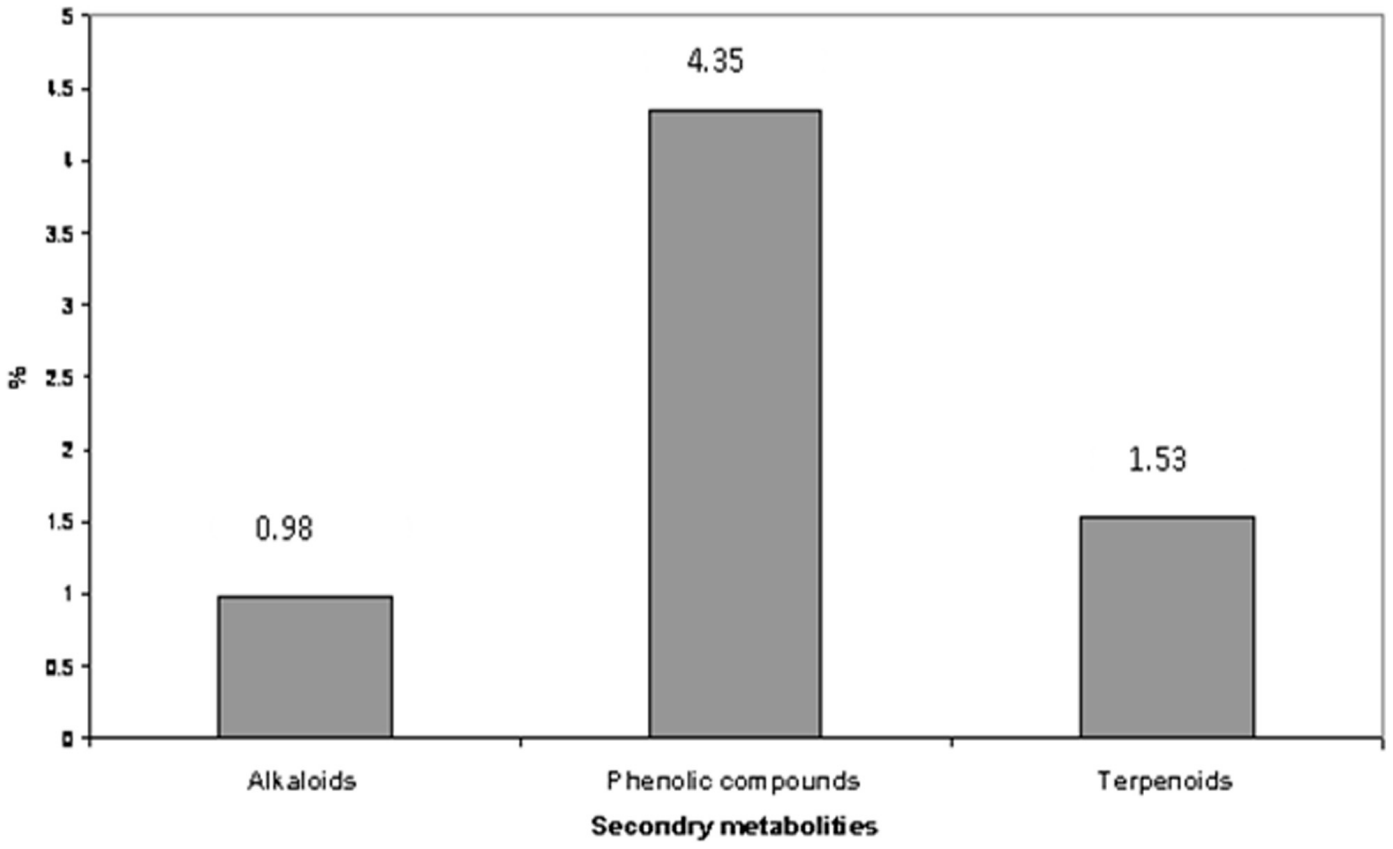
Phytochemical analysis of major secondary metabolites present in methanolic extract of *Eichhornia crassipes*.

Fraction A (with R_f_ 0.47, M. Wt, 352) of the crude extract was identified by spectroscopic methods as an alkaloid (18, 19-Seco-15 beta-yohimb) and this is the first time for separation of this compound from water hyacinth and was shown to exert potent antibacterial, antialgal and moderate antifungal activities (Table 1 and 2). This fraction was not detected in GC/MS of the crude extract K (Table 3).The fractionation of this compound may be explained by a possible interaction between certain phthalate backbone [skeleton] and the acetamide derivatives or other nitrogenous compounds, recorded in GC/MS of the crude extract (Table 3). The fraction may have formed during the extraction and fractionation processes. The mass spectrum of active compounds separated from methanolic extract of *E. crassipes* showed that all compounds share the common fragment ion as the following: 57, 71, 85, 149, 167, 207 and 279 Da as shown in Fig. 3 and these results were confirmed by H-NMR. The NMR data indicated that all fractions had the following types of protons ; A multiplex signal at δ 6.89–7.47 ppm was characteristic of aromatic protons; the singlet signal at δ 5.320 ppm was characteristic of the two protons of olifinic (CH = CH); the singlet signal at δ 3.342 ppm was characteristic of four protons of two O-CH_2_ group; the singlet signal at δ 1.26, 1.6 and 2.5 ppm was characteristic of the protons of methylene (CH_2_) group and the singlet signal at δ 1.9 ppm was characteristic of protons of methyl (CH_3_) group]. However, it was recorded from the phytochemical analysis of the crude extract which contained 0.98% alkaloids, 4.35% phenolic compounds and 1.53% terpenoids as illustrated in Fig. 4. The minimum inhibitory concentration (MIC) of Fraction A (Table 4) which inhibited the growth of different bacterial, fungal and algal species was ranged between 20 µg/ml (in case of *C. vulgaris*) to 95 µg/ml (in case of *C. albicans*).

**Table 3:** GC/MS of crude methanolic extract of *Eichhornia crassipes*.

Peak number	Rt	Compound	Relative percentage (%)
1	3.15	1-Deuterio-trans-1,3 dihydroxy	8.03
2	5.09	(E)-4-acetoxy-2-pentenenitrile	2.13
3	8.59	Trans-2-Tridecenal	2.21
4	8.90	3(2H)-Isothiazolone, 2-methyl	2.97
5	9.97	9-Hexadecenoic acid	2.49
6	10.51	Urox	2.83
7	11.73	Silane	6.84
8	11.77	Isopropylimino dibenzyl	4.61
9	12.01	13-oxabicyclo (10.1.0)tridecane	2.76
10	12.23	2-dodecenoic acid	2.26
11	12.89	Androsta-3, 5-dien-3-ol, 3-O-dimethyl	10.84
12	13.86	1,2-Benzenedicarboxylic acid	3.63
13	14.15	Eicosamethylcyclodecasiloxane	3.87
14	15.67	Benzeneacetic acid	3.58
15	16.43	Sotalol	2.62
16	16.66	Phosphonic acid	3.25
17	16.89	Fenuron	2.16
18	17.17	10-Undecenoic acid	2.13
19	17.43	Hydantoin, 1-N-Formyl-5-hydroxy	3.72
20	17.54	Cyclohexanone	5.27
21	17.64	Butanoic acid	4.27
22	17.68	N, N-diacetyl-1,7-diaminoheptane	3.92
23	17.72	N-(3-methylbutyl)-acetamide	3.20
24	19.66	Urea	2.27
25	20.07	Alpha-D-Xylofuranoside	2.48
26	20.15	9-Octadecenoic acid (Z)	3.46
27	20.66	3-Tidecanone	2.19

*Rt: Retention time.

**Table 4:** Minimum inhibition concentration (MIC) of active fraction (A) from *Eichhornia crassipes*.

Test organisms	MIC (µg/ml)
*Bacillus subtilis*	92
*Escherichia coli*	78
*Staphylococcus aureus*	76
*Streptococcus faecalis*	55
*Candida albicans*	95
*Chlorella* vulgaris	20
LSD at 0.01	0.26

*Each value is presented as mean of triplicate treatments, LSD: Least different significantly at p≤0.01 according to Duncan's multiple range test. Data are expressed as mean ± S.D.

The obtained results could be concluding that, Water hyacinth was shown to be an abundant source of new and useful antibiotics active against some pathogenic strains of bacteria, fungi and algae. The active compounds were complex in structure and so would be difficult and expensive to synthesize chemically. Controlling the wide spread of the water hyacinth in the different Egyptian bodies (River Nile and its canals) may be achieved by harvesting it for pharmaceutical uses. Extracts and fractions used pharmaceutically could require the harvest of millions of tones/year.
